# Serum Uric Acid and Chronic Kidney Disease: The Role of Hypertension

**DOI:** 10.1371/journal.pone.0076827

**Published:** 2013-11-12

**Authors:** Sanaz Sedaghat, Ewout J. Hoorn, Frank J. A. van Rooij, Albert Hofman, Oscar H. Franco, Jacqueline C. M. Witteman, Abbas Dehghan

**Affiliations:** 1 Department of Epidemiology, Erasmus Medical Center, Rotterdam, The Netherlands; 2 Department of Internal Medicine—Nephrology, Erasmus Medical Center, Rotterdam, The Netherlands; University of Sao Paulo Medical School, Brazil

## Abstract

**Background:**

There are inconsistent findings on the role of hyperuricemia as an independent risk factor for chronic kidney disease (CKD). Hypertension has been implicated as a factor influencing the association between serum uric acid and CKD. In this population-based study we investigated the association between serum uric acid and decline in renal function and tested whether hypertension moderates this association.

**Methods:**

We included 2601 subjects aged 55 years and over from the Rotterdam Study. Serum uric acid and estimated glomerular filtration rate (eGFR) were assessed at baseline. After average 6.5 years of follow-up, second eGFR was assessed. CKD was defined as eGFR<60 ml/min/1.73 m^2^. All associations were corrected for socio-demographic and cardiovascular factors.

**Results:**

Each unit (mg/dL) increase in serum uric acid was associated with 0.19 ml/min per 1.73 m^2^ faster annual decline in eGFR. While the association between serum uric acid and incidence of CKD was not significant in our study population (Hazard Ratio: 1.12, 95% confidence interval [CI]: 0.98–1.28), incorporating our results in a meta-analysis with eleven published studies revealed a significant association (Relative Risk: 1.18, 95%CI: 1.15–1.22). In the stratified analyses, we observed that the associations of serum uric acid with eGFR decline and incident CKD were stronger in hypertensive subjects (P for interaction = 0.046 and 0.024, respectively).

**Conclusions:**

Our findings suggest that hyperuricemia is independently associated with a decline in renal function. Stronger association in hypertensive individuals may indicate that hypertension mediates the association between serum uric acid and CKD.

## Background

The incidence of chronic kidney disease (CKD) is steadily increasing [1]. Affected individuals have high rates of cardiovascular morbidity and mortality and often require costly treatments such as dialysis and kidney transplantation [2]. Identifying novel risk factors for CKD may improve preventative programs that would eventually decrease the burden caused by this disease [3].

One of the recently proposed risk factors for CKD is hyperuricemia [4]. Although elevated serum uric acid is associated with CKD, it is not clear whether hyperuricemia plays a detrimental role in developing CKD or if it merely is a consequence of lower glomerular filtration rate prior to CKD [5]. To this end, different prospective studies investigated the association between serum uric acid and incident CKD [4], [6]–[18]. However, these studies showed inconsistent findings [19]–[21]. Furthermore, a growing body of evidence supports a role for uric acid in developing hypertension, a well-established risk factor for CKD [22], [23]. We hypothesize that hypertension might mediate the effect of uric acid on renal function. If so, the association between serum uric acid and CKD should be stronger in hypertensive individuals.

In this study we investigated the association of serum uric acid with decline in estimated glomerular filtration rate (eGFR) and incident CKD in the Rotterdam Study, a prospective cohort study of individuals 55 years and older. Moreover, we performed a meta-analysis to provide a reliable estimate of the effect of serum uric acid on risk of CKD. Finally, we studied whether this association differs in hypertensive and normotensive individuals.

## Methods

### Population

The Rotterdam Study is a population-based cohort study, including 7,983 participants living in Ommoord, a district of Rotterdam, The Netherlands. All participants aged 55 and over, were invited to this study (n = 10,275). The Rotterdam Study started in the early 1990s and periodical examinations were performed every 3 to 5 years. In addition, participants were continuously followed for vital status, obtaining information regularly from the municipal health authorities in the Rotterdam area. The study was approved by the Medical Ethics Committee of the Erasmus Medical Center and written informed consent was obtained from all participants [24], [25].

### Uric acid

Serum uric acid was measured once at baseline. Values of serum uric acid were obtained from baseline non-fasting blood samples which were centrifuged for 10 minutes at 3000 rotations per minute and subsequently stored at −20°C for one week. Uric acid activity was ascertained with Kone Diagnostica reagent kit and Kone autoanalyzer [26]. In order to check the calibration, for every 10 samples, 3 control samples were included. In each run (100 samples), if the average values of the control samples were not within 2.5% of the true value, the run was repeated. Calibration was also done on day-by-day variation, which had to be within 5% [27].

### eGFR decline and incident CKD

Serum creatinine was measured twice; first at baseline visit and second at follow up visit. At baseline visit serum creatinine was determined using non-kinetic alkaline picrate (Jaffé) method. At follow up visit serum creatinine was determined using an enzymatic assay method [28]. In order to calibrate, we aligned the mean values of serum creatinine with serum creatinine values of the participants of the Third National Health and Nutrition Examination Survey (NHANES III) in different gender and age groups (<60, 60–69, ≥70). Measurements were done for 5280 individuals at baseline (1989–1993) and 3867 individuals at the follow up visit (1997–1999). eGFR was calculated using the simplified Modification of Diet in Renal Disease (MDRD) equation which is recommended by the National Kidney Foundation [28]. CKD was defined as eGFR<60 ml/min per 1.73 m^2^. To calculate the annual eGFR decline, we first subtracted the eGFR estimates of the follow up examination from the eGFR estimates at baseline and then divided by the time between the two visits. These two examinations were on average 6.5 years apart. Incident cases were defined among the individuals free of CKD at baseline (eGFR>60 ml/min per 1.73 m^2^)_,_ who had a decline in eGFR to less than 60 ml/min per 1.73 m^2^ between the two periodical examinations. To estimate the censoring date of the cases, we assumed a linear decrease in eGFR. Given this assumption, the date that each case had passed the eGFR threshold of 60 ml/min per 1.73 m^2^ was taken as the censoring date and it was used to calculate the follow up time for incident cases. For controls, the time spent between the two examinations was used as the follow up time.

### Covariates

Body mass index was calculated by dividing weight in kilograms by height in meters squared. Serum total cholesterol and high density lipoprotein cholesterol levels was determined using an automated enzymatic method. Information on smoking and alcohol consumption was acquired from the questionnaires. Participants were asked for the average daily consumption of alcohol. Coronary heart disease was considered as experiencing myocardial infarction or coronary revascularization procedures. Diabetes mellitus was defined as the use of blood glucose lowering drugs or a random non-fasting glucose above 11.1 mmol/l. Hypertension was defined as systolic blood pressure ≥140 mmHg or diastolic blood pressure ≥90 mmHg or use of blood pressure lowering medication with hypertension as the indication. Medication use information is based on home interview. Data for all the covariates were obtained once at baseline.

### Population for analysis

**Figure 1:**
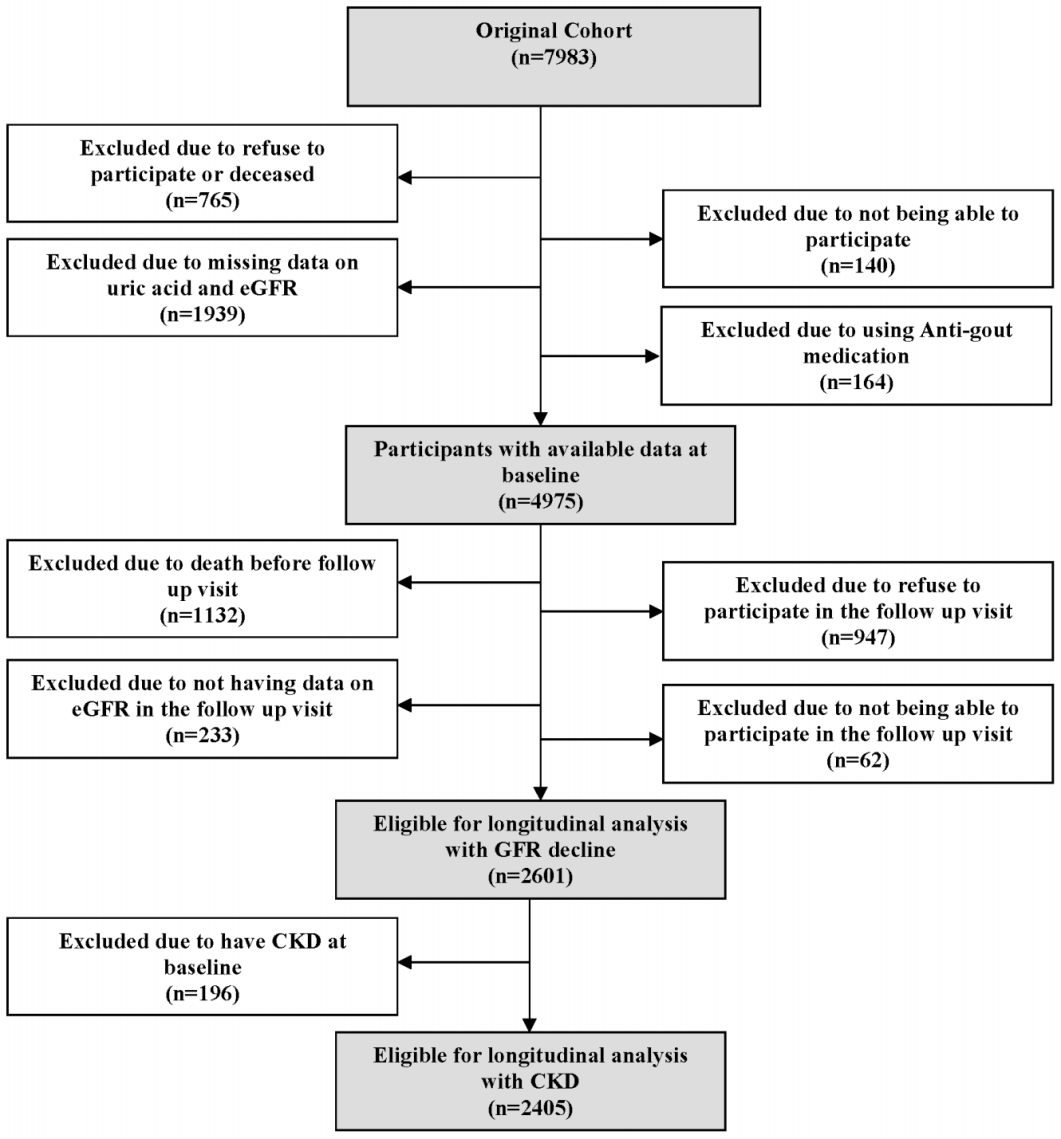
Population for analysis.

As depicted in Figure 1, from 7983 participants at baseline, 5139 individuals had available data at baseline. We further excluded 164 participants using antigout medications (allopurinol, probenecid, benzbromarone, and colchicine). Among 4975 subjects 1132 died during the 6.5 years of follow-up time, 62 participants were not able to participate in the follow up visit, 947 subjects did not participate in the follow up visit and 233 did not have second serum creatinine measurements. This resulted in a sample of 2601 participants for the longitudinal analyses on eGFR decline. Furthermore, we excluded subjects with baseline CKD (n = 196) for the analyses on the incidence of CKD.

### Statistical analysis

A linear regression model was used to evaluate the association between serum uric acid (mg/dL) and eGFR decline. The Cox proportional hazard model was applied to calculate the hazard ratio (HR) for the association between serum uric acid and incidence of CKD. All analyses were adjusted for age, sex and baseline eGFR. In a multivariate analysis, we additionally adjusted for potential confounders including systolic blood pressure, diabetes mellitus, body mass index, high density lipoprotein, alcohol consumption, smoking, total cholesterol, coronary heart disease, and use of diuretics, beta blockers, calcium channel blockers, and ACE inhibitors. Analysis was further done in subgroups of hypertensive and normotensive individuals. Interaction was assessed, by adding an interaction term in the regression model. The interaction term was the product of the interacting factor and serum uric acid. R version 2.13.0 was used to calibrate creatinine values at baseline and follow up. All the other analyses were carried out using SPSS 17.0.2 for windows.

### Meta-analysis

We searched for studies published in MEDLINE (PubMed), EMBASE and Web of Science using the common key words related to incidence of chronic kidney disease and serum uric acid including “renal disease” or “renal insufficiency” or “kidney disease” and “blood urate” or “serum uric acid” or “hyperuricemia”. We restricted the language of the search to English. Population-based studies which evaluated the association between serum uric acid and incidence of CKD were included in our meta-analysis. Some of the eligible studies had used different outcome definitions and measurements of serum uric acid. Therefore, we contacted nine authors to obtain results consistent with our definitions and adjustments. Authors were asked to adjust the analysis for the following variables: age, sex, smoking, alcohol consumption, body mass index, diabetes, hypertension, total cholesterol, baseline kidney function, and proteinuria. From 12 studies, 6 studies provided HR and 6 studies used odds ratio (OR) to report the effect size. Since the incidence of CKD is relatively low [29], we accepted OR as a proxy for HR and combined them in the meta-analysis. Data used for the meta analysis is available in supplementary document (Table S1).The heterogeneity assumption was investigated using a commonly used statistical method, namely the I-square statistic [30]. Publication bias was evaluated using the Egger's test [31]. The statistical analyses were performed using the “meta” package of the statistical software R, version 2.13.0. Moreover, we tested the publication bias using STATA version 10.

## Results

As depicted in Figure 1 among 4975 individuals at risk of developing CKD at baseline, 2374 subjects either died or were lost to follow up during 6.5 years of follow up. Comparing them with the population included in the analysis, they were significantly older and had higher C-reactive protein level, systolic blood pressure, and serum uric acid. They also had lower eGFR, alcohol consumption, total cholesterol and body mass index. Finally, they were more likely to use antihypertensive medication and to have diabetes mellitus, CKD, and coronary heart disease (Table S2).

### Baseline characteristics

The study population had an average age of 70.4 years, serum uric acid level of 5.38 mg/dL, and eGFR of 77.15 ml/min per 1.73 m^2^. As shown in Table 1, subjects in the higher quartiles of serum uric acid were older and more likely to be male, diabetic, alcohol drinker, and former smoker. They had a significantly higher body mass index, systolic and diastolic blood pressure, C-reactive protein level, history of coronary heart disease and prevalence of antihypertensive medications use. In addition, they had lower levels of eGFR and HDL cholesterol.

**Table 1:** Baseline characteristics of the participants in different quartiles of uric acid levels.

	Uric Acid Quartiles (mg/dL*)				
	≤4.5	4.5–5	5–6	>6	P for trend**
	(n = 1257)	(n = 1210)	(n = 1263)	(n = 1245)	
**Age, mean (SD** † **), y**	69.1(8.9)	70.4(9.2)	70.4(9.0)	71.5(9.4)	<0.001
**Men (%)**	208 (16.5)	374(30.9)	586(46.4)	705(56.6)	<0.001
**Smoking**					
**Current (%)**	312(24.8)	277(22.9)	313(24.8)	245(19.7)	0.001
**Former (%)**	366(29.1)	429(35.5)	529(41.9)	614(49.3)	
**Daily Alcohol Intake in drinkers, median (Interquartile range), g/d**	4.5(0.7–13.6)	5.0(1.0–14.8)	9.3(1.7–21.2)	11.2(2.5–27.9)	<0.001
**Body mass index, mean (SD), kg/m^2^**	25.2(3.4)	25.7(3.5)	26.6(3.6)	27.3(3.8)	<0.001
**Waist circumference, mean (SD), cm**	84.8(10.8)	87.7(10.3)	91.7(10.2)	94.8(10.5)	<0.001
**Total cholesterol, mean (SD), mmol/L**	6.6(1.2)	6.5(1.2)	6.6(1.3)	6.6(1.2)	0.472
**HDL cholesterol, mean (SD), mmol/L**	1.4(0.3)	1.3(0.3)	1.3(0.3)	1.2(0.3)	<0.001
**C-reactive protein, median (Interquartile range), mg/L**	1.3(0.6–2.7)	1.8(0.9–3.4)	2.0(1.0–3.8)	2.5(1.2–4.6)	<0.001
**Glomerular filtration rate, mean (SD), ml/min per 1.73 m2**	82.9(16.8)	78.3(15.4)	76.1(16.3)	71.1(18.2)	<0.001
**Systolic blood pressure, mean (SD), mm Hg**	137.3(22.2)	138.3(21.7)	139.3(21.5)	141.2(22.4)	<0.001
**Diastolic blood pressure, mean (SD), mm Hg**	72.6(11.5)	72.8(11.6)	73.4(11.6)	74.0(12.1)	0.091
**Chronic kidney disease (%)**	65(5.2)	116(9.6)	173(13.7)	331(26.6)	<0.001
**Diabetes Mellitus (%)**	136(10.9)	109(9.1)	125(9.9)	178(14.4)	<0.001
**History of coronary heart disease (%)**	88(7.1)	121(10.2)	193(15.4)	264(21.6)	<0.001
**Diuretics (%)**	101(8.0)	125(10.3)	189(15.0)	413(33.2)	<0.001
**Calcium channel blockers (%)**	52(4.1)	46(3.8)	75(5.9)	131(10.5)	<0.001
**Beta-blockers (%)**	103 (8.2)	134(11.1)	192(15.2)	293(23.5)	<0.001
**ACE inhibitors (%)**	41(3.3)	33(2.7)	61(4.8)	126(10.1)	<0.001

* To convert to SI unit multiply by 59.48.

** P-value adjusted for age and sex for continuous measure of uric acid.

† Standard Deviation.

### Serum uric acid and eGFR decline

**Figure 2:**
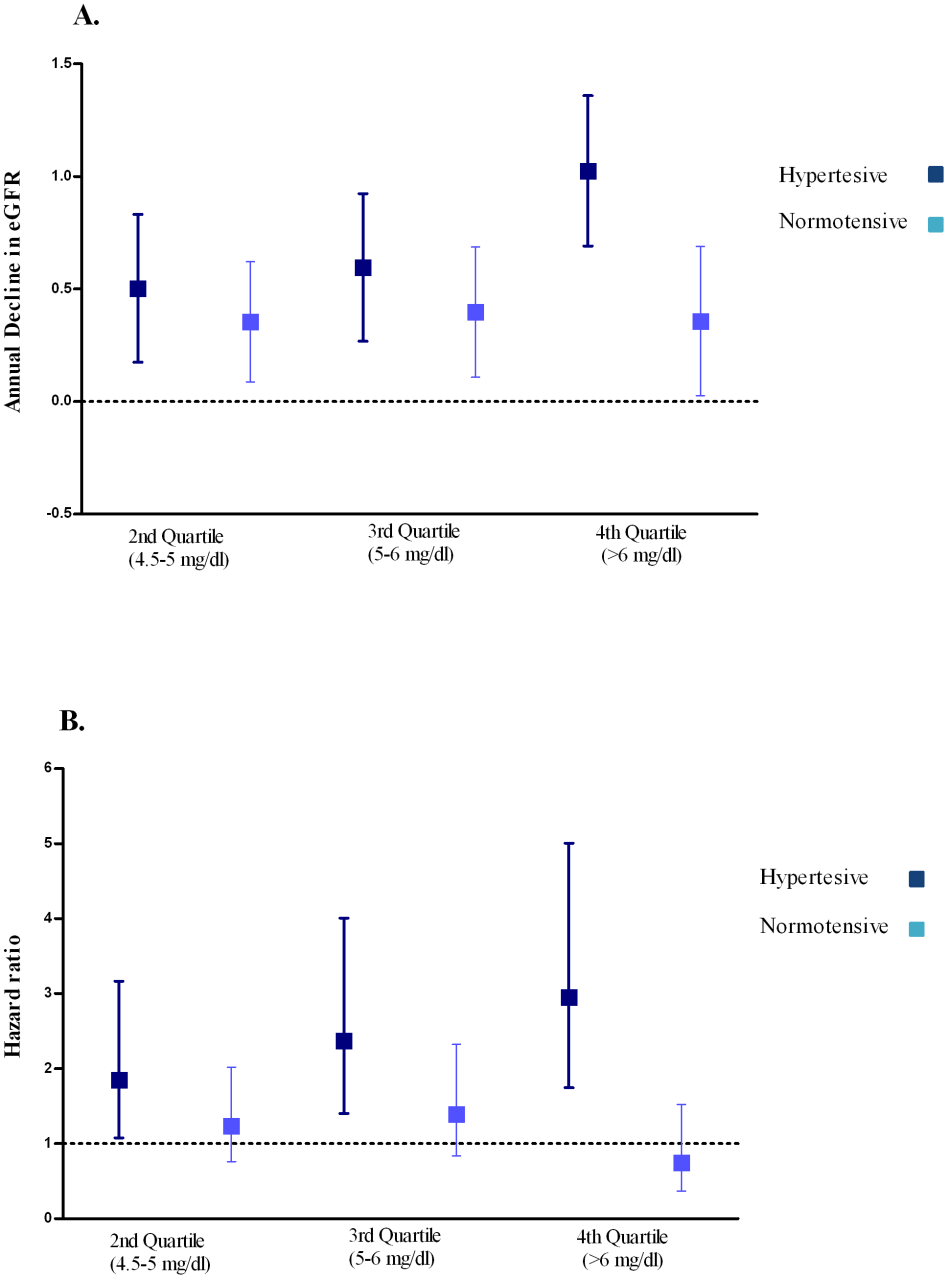
Association of serum uric acid with kidney function in normotensive and hypertensive subjects (A) Annual decline in eGFR in relation to serum uric acid quartiles in hypertensive and normotensive participants. Analyses are adjusted for age, sex and baseline eGFR. Quartiles are compared with participants in the first quartile of serum uric acid (<4.5 mg/dL) (**B**) Risk of incident CKD in relation to quartiles of serum uric acid level in hypertensive and normotensive participants. Analyses are adjusted for sex, age and baseline eGFR. All odds ratios are compared with participants in the first quartile of serum uric acid (<4.5 mg/dL).

Average annual eGFR decline was 0.92 ml/min per 1.73 m^2^ (SD = 2.21) for all participants. For each unit increase in serum uric acid, the annual eGFR decline was higher by 0.19 ml/min per 1.73 m^2^ (95% confidence interval [CI]: 0.13–0.26) in the age and sex adjusted model and 0.18 ml/min per 1.73 m^2^ (95%CI: 0.10–0.26) in the multivariate adjusted model (Table 2). We repeated the analyses in subgroups of hypertensive and normotensive participants. In both models, the association was stronger in hypertensive subjects compared to normotensive subjects (P-value for interaction = 0.020). Hypertensive participants in the highest quartile of serum uric acid had 1.12 higher risk of annual eGFR decline per one unit increase in serum uric acid compared to those in the lowest quartile (Figure 2-A).

**Table 2:** The association between serum uric acid (mg/dL) and decline in estimated glomerular filtration rate (ml/min per 1.73 m^2^).

	Minimally adjusted Model**				Multivariate adjusted Model†			
	*N*	*Annual decline*	*95% CI* *	*P-value*	*N*	*Annual decline*	*95% CI*	*P-value*
**Total population**	2601	0.19	0.13, 0.26	1.2×10^−10^	2312	0.18	0.10, 0.26	1.0×10^−5^
**Normotensive**	1312	0.13	0.03, 0.23	0.008	1167	0.14	0.03, 0.25	0.014
**Hypertensive**	1275	0.24	0.14, 0.33	7.8×10^−9^	1145	0.20	0.08, 0.31	0.001
**P for interaction**				0.020				0.046

* CI: confidence interval.

** Adjusted for age, sex, and baseline eGFR.

† Adjusted for age, sex, systolic blood pressure, body mass index, alcohol consumption, smoking, high density lipoprotein, diabetes mellitus, coronary heart disease, total cholesterol, and the use of diuretics, beta blockers, calcium channel blockers, ACE inhibitors, and baseline eGFR.

### Serum uric acid and incidence of CKD

As shown in Table 3, serum uric acid was associated with incident CKD in age and sex adjusted model (HR: 1.22; 95%CI: 1.10–1.35). After further adjusting for potential confounders, the direction of the association remained the same; however, the strength of the association attenuated and it was no longer statistically significant (1.12; 95%CI: 0.98–1.28). We further examined the association between serum uric acid and CKD separately in hypertensive and normotensive participants. In both models, the association was present in hypertensive subjects (HR: 1.29, 95%CI: 1.14–1.46) but absent in normotensive subjects (HR: 1.03, 95%CI: 0.85–1.24) (P-value for interaction = 0.030). Hypertensive participants in the highest quartile of serum uric acid had more than three times higher risk of developing CKD compared with those in the lowest quartile (Figure 2-B). To further explore whether other metabolic factors, including high density lipoprotein, diabetes, and waist circumference, could influence the association of uric acid with incidence of CKD, we performed a series of stratified analyses. These stratified analyses showed that there was no statistical difference, in the association of serum uric acid and incidence of CKD, between different groups of participants (Figure S1).

**Table 3:** The association between serum uric acid (mg/dL) and incidence of chronic kidney disease.

	Minimally adjusted Model†				Multivariate adjusted Model††			
	*N(case)*	*HR* *	*95% CI* **	*P-value*	*N(case)*	*HR* *	*95% CI*	*P-value*
**Total population**	2405 (289)	1.22	1.10, 1.35	1.8×10^−2^	2154 (249)	1.12	0.98, 1.28	0.079
**Normotensive**	1243 (112)	1.03	0.85, 1.24	0.747	1111(102)	0.96	0.78,1.19	0.758
**Hypertensive**	1149 (175)	1.29	1.14, 1.46	4.4×10^−3^	1043(147)	1.23	1.04, 1.45	0.016
**P for interaction**				0.030				0.024

* HR: hazard ratio.

** CI: confidence interval.

† Adjusted for age, sex, and baseline eGFR.

†† Adjusted for age, sex, systolic blood pressure, body mass index, alcohol consumption, smoking, high density lipoprotein, diabetes mellitus, coronary heart disease, total cholesterol, diuretics, beta blockers, calcium channel blockers, ACE inhibitors, and baseline eGFR.

### Meta-analysis of serum uric acid and incidence of CKD

**Figure 3:**
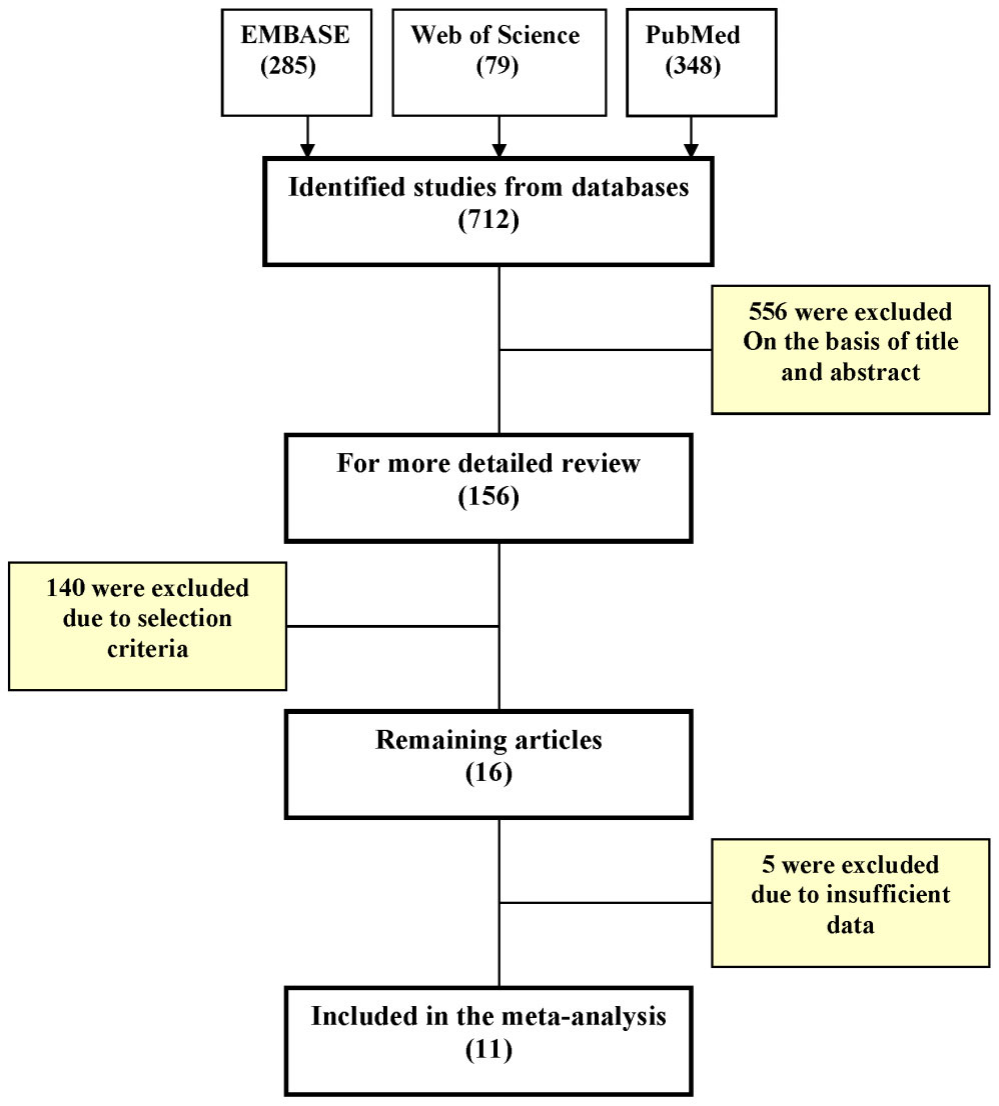
Flow diagram of studies through the different phases of the meta-analysis.

**Figure 4:**
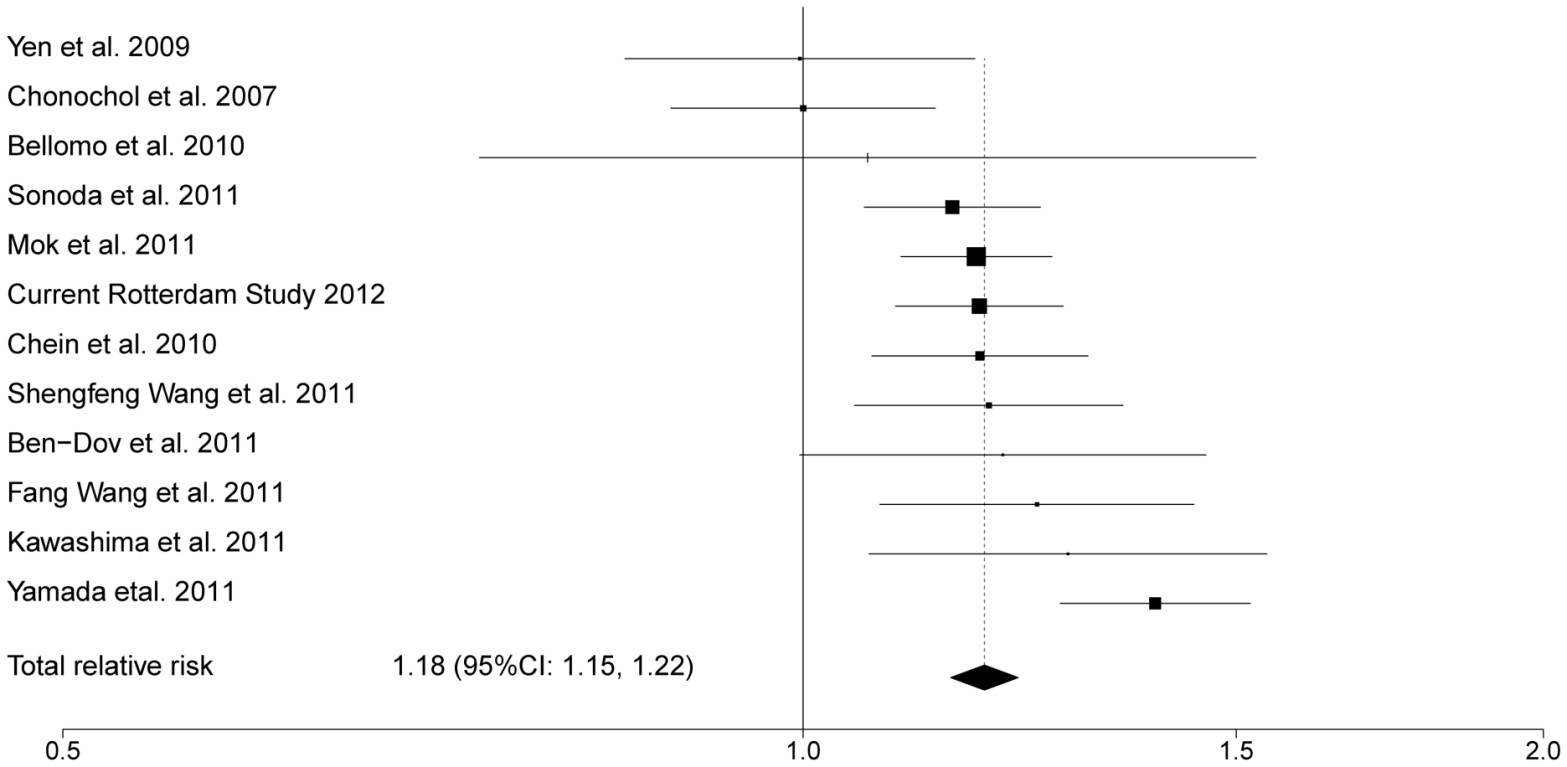
Forest plot of multivariate adjusted relative risk for CKD incidence associated with continuous values of serum uric acid (mg/dL).

Figure 3 shows the flow diagram for inclusion of the relevant studies in our meta-analysis [6], [7], [12], [17], [18], [32]–[37]. We performed a fixed effect model meta-analysis on 12 studies including 11 published studies and the current analysis from the Rotterdam Study (Figure 4). The overall relative risk was 1.18 (95%CI: 1.15–1.22) for incidence of CKD per 1 mg/dL higher level of serum uric acid. There was no evidence of publication bias (Egger's test P-value = 0.583) and test for heterogeneity resulted in moderate estimates (I^2^ = 57.7% [19.7%–77.7%]).

## Discussion

In this prospective cohort study, we found that high levels of serum uric acid are associated with faster decline in eGFR and increased incidence of CKD. Moreover, combining our finding with 11 published population-based studies in a meta-analysis suggested a role for serum uric acid as an independent predictor of incident CKD. Finally, we observed that the association is more pronounced in hypertensive subjects compared to normotensive individuals.

A number of recent studies evaluated the relation between serum uric acid and incidence of CKD [4], [7]–[17], [38]. In agreement with our findings, some of them introduced high serum uric acid as a risk factor for the development of CKD [4], [6]–[13], [17]. Weiner et al. pooled data from Atherosclerosis Risk in Communities (ARIC) study and Cardiovascular Health Study (CHS) and found an odds ratio of 1.11 for CKD incidence per one unit (mg/dl) increase in serum uric acid [13]. However, these findings were contradicted with some other reports. A large cohort of 28,745 young participants (age 20–49 years) in Taiwan showed a weak correlation between serum uric acid and eGFR (Pearson correlation = −0.22) [20]. Moreover, Sturm et al. analyzed 227 patients with primary non-diabetic CKD and found that the association between high serum uric acid and progression of CKD disappeared after adjustment for potential confounders [21]. Given these inconsistencies, we performed a meta-analysis to combine the results of published studies on serum uric acid and incident CKD. Our meta-analysis, based on 12 studies including the current study, confirmed the association between high serum uric acid and CKD incidence. This finding is in accordance with the results of trials that have shown a slower progression of CKD after treatment with allopurinol [39], [40].

We found evidence for moderate heterogeneity in the association between serum uric acid and CKD in our meta-analysis. One possible explanation is that potential intermediate factors are not evenly distributed among these studies. Hypertension is one of the factors that may play an important role in the association between serum uric acid and CKD. The correlation between serum uric acid and hypertension is well-documented [41], [42] and a large number of studies have reported linear and dose dependent associations between serum uric acid and Blood pressure. In current study we observed that the association between high serum uric acid and CKD was stronger in hypertensive individuals compared to normotensives. In agreement with this finding, in the study of normotensive adults no association was found between high serum uric acid and incidence of CKD [7]. These findings may offer an explanation for the heterogeneity between studies and may provide evidence for possible role of hypertension in mediating the association between high serum uric acid and CKD.

Different explanations can be presented for our observation regarding the association of serum uric acid with risk of CKD and the role of hypertension. Uric acid can increase the risk of CKD directly through inhibition of endothelial nitric oxide bioavailability, activation of the renin-angiotensin system, and increase in renal microvascular damage [43], [44]. Moreover, it is possible that high serum uric acid leads to kidney damage through conventional risk factors for CKD such as hypertension. It has been shown that high serum uric acid increases the risk of developing hypertension by enhancing salt sensitivity [23]. Another possible explanation might be that antihypertensive medications such as diuretics increase serum uric acid level in hypertensive individuals [45], and consequently elevated serum uric acid may directly result in kidney damage [46]. In this study we adjusted our analyses for different types of antihypertensive medications including diuretics; therefore it is unlikely that the associations were influenced by the medication use. We adjusted the analyses for the use of any diuretic; however, we acknowledge that different types of diuretics have differential impact on serum uric acid concentrations.

Our study has several strengths. First, the Rotterdam Study is a large population-based cohort study, which on one hand provides sufficient statistical power to answer our research question and on the other hand could be generalized to general population. Second, we controlled for several potential confounders such as different types of antihypertensive medications, and performed the analyses separately in subgroups of hypertensives and normotensives. Third, we performed a meta-analysis that provides a robust estimate of the association. Different limitations of this study should also be acknowledged. First, no data on albuminuria were available, which is an important element in defining CKD. However, CKD definition of eGFR<60 ml/min per 1.73 m^2^ is a well-accepted definition in population-based research settings [47]. Second, 2374 participants were lost to follow up, mainly because they died before the follow up visit. These subjects were older, had lower eGFR, higher serum uric acid level, and more often had hypertension and diabetes mellitus. Since subjects who dropped out from our study during follow up period had higher uric acid levels and were more likely to develop CKD, we may have underestimated the association between serum uric acid and risk of CKD.

### Conclusion

We have demonstrated that serum uric acid is independently associated with the risk of CKD. This association was significantly stronger in hypertensive individuals. Future studies are needed to test whether better monitoring or even lowering of serum uric acid levels in hypertensive patients can slow down the progression of CKD.

## Supporting Information

Figure S1 **Association of serum uric acid and incidence of CKD in different subgroups of participants with and without components of metabolic syndrome.**
(DOCX)

Table S1 **Characteristics of the included studies in the meta-analysis on serum uric acid and incidence of CKD.**
(DOCX)

Table S2 **Comparing baseline characteristic of population for analysis with missing population (died or loss to follow up).**
(DOCX)
