# Supplementary material for: Serum Uric Acid and Chronic Kidney Disease: The Role of Hypertension
Source: PLoS One. 2013 Nov 12;8(11):e76827. doi: 10.1371/journal.pone.0076827 (PMC3827035; doi:10.1371/journal.pone.0076827)
Supplement: Figure S1 — Association of serum uric acid and incidence of CKD in different subgroups of participants with and without components of metabolic syndrome. (DOCX) [file pone.0076827.s001.docx]

**Figure S1.** Association of serum uric acid and incidence of CKD in different subgroups of participants with and without components of metabolic syndrome

Abbreviations; HR: Hazard ratio, HDL: High density lipoprotein cholesterol, WC: Waist circumference

* (Definition Low HDL: <1.0 mmol/L for men; <1.3 mmol/L for women, and High HDL >=1.0 mmol/L for men; >=1.3 mmol/L for women)

**(Definition High WC: >=102 cm for men; >=88 cm for women, and Low WC: < 102 cm for men; < 88 cm for women ) [[1](#_ENREF_1)]

**References**

1. Alberti KG, Eckel RH, Grundy SM, Zimmet PZ, Cleeman JI, et al. (2009) Harmonizing the metabolic syndrome: a joint interim statement of the International Diabetes Federation Task Force on Epidemiology and Prevention; National Heart, Lung, and Blood Institute; American Heart Association; World Heart Federation; International Atherosclerosis Society; and International Association for the Study of Obesity. Circulation 120: 1640-1645.
