# Supplementary material for: Serum Uric Acid and Chronic Kidney Disease: The Role of Hypertension
Source: PLoS One. 2013 Nov 12;8(11):e76827. doi: 10.1371/journal.pone.0076827 (PMC3827035; doi:10.1371/journal.pone.0076827)
Supplement: Table S1 — Characteristics of the included studies in the meta-analysis on serum uric acid and incidence of CKD. (DOCX) [file pone.0076827.s002.docx]

**Table S1:** Characteristics of the included studies in the meta analysis on serum uric acid and incidence of CKD

| **First Author, year** | **Population**  **(Sample size)** | **Measure of association** | **Outcome** | **Adjusted covariates** | **Effect estimate**  **(95%CI)** |
| --- | --- | --- | --- | --- | --- |
| Yen et al. 2009 | Health check up  (800) | OR | CKD:eGFR less than 60 | Age, sex, smoking, BMI, DM, HTN, Chol, Albumin, BUN, Cr, Proteinuria, Hgb, WBC, plt | 0.99 (0.84,1.17) |
| Chonochol et al. 2007 | Population based  (4800) | OR | CKD:eGFR less than 60 | Age, sex, race, Cr, BMI, WC, SBP, DBP, Antihypertensive medication, BG, HDL, TG, Ankle-arm index, IMT, Hgb, CRP, Alb | 1.00 (0.89, 1.14) |
| Bellomo et al. 2010 | Normotensive blood donors  (900) | HR | CKD:eGFR less than 60 | Age, sex, smoking, alcohol drinking, BMI, DM, HTN, Chol, proteinuria, Cr baseline | 1.06 (0.74, 1.54) |
| Sonoda et al. 2011 | Health check up (7078) | OR | CKD:eGFR less than 60 | BMI, SBP, FBS, HDL, LDL, Hgb, Smoking, eGFR | 1.15(1.06, 1.25) |
| Rottedam Study 2011 | Population based (2601) | HR | CKD:eGFR less than 60 | Age, sex, SBP, BMI, alcohol consumption, smoking, HDL, DM, CHD, Chol, diuretics, beta blockers, calcium channel blockers, ACE inhibitors, baseline eGFR | 1.12 (0.98,1.28) |
| Chein et al. 2010 | Health check up (5168) | HR | CKD:eGFR less than 60 | Age, sex, DBP, History of stroke, BS, HbA1c, Proteinuria | 1.18 (1.07, 1.31) |
| Mok et al. 2011 | Population based  (14939) | HR | CKD:eGFR less than 60 | Age, smoking status, alcohol, exercise, BMI, Chol, HTN, DM | 1.17(1.09, 1.26) |
| Shengfeng Wang et al. 2011 | Heath check up  (7488) | OR | CKD:eGFR less than 60 | Age, sex, education, alcohol drinking, smoking, exercise, BMI, TG, Chol, LDL, HDL, albumin, CRP, BUN, Hgb, Hct, eGFR, proteinuria, hematuria, HTN, DM, medication | 1.19 (1.05, 1.35) |
| Ben-Dov et al. 2011 | Population based (2449) | HR | Hospital diagnosis of CKD | Age, sex, smoking, alcohol drinking, BMI, DM, HTN, Chol, proteinuria, Cr baseline | 1.20 (0.66, 1.46) |
| Fang Wang et al. 2011 | Population based (1563) | OR | CKD:eGFR less than 60 | Age, sex, BMI, history of CHD, smoking, DM, SBP, TG, HDL, Albuminuria, eGFR | 1.25 (1.10, 1.43) |
| Kawashima et al. 2011 | Male workers (1285) | HR | CKD:eGFR less than 60 | Age, sex, smoking, alcohol drinking, BMI, DM, HTN, Chol, proteinuria, eGFR | 1.28 (1.06, 1.54) |
| Yemada, et al. 2011 | Heath check up  (14399) | OR | CKD:eGFR less than 60 | Age, BMI, HTN, hypertriglyceridemia, FBS, urinary occult blood or protein or both, alcohol drinking, smoking | 1.39 (1.27, 1.52) |

Abbreviations; BMI: Body mass index, HDL: High density lipoprotein cholesterol, LDL: Low density lipoprotein cholesterol WC: Waist circumference, DM: Diabetes mellitus, Chol: Cholesterol, HTN: Hypertension, Cr: Creatinine, Hgb: Hemoglobin, WBC: White blood cell count, Plt: Platelet, SBP: Systolic blood pressure, DBP: Diastolic blood pressure, BG: Blood glucose, TG: Triglyceride, IMT: Intima media thickness, CRP: C-reactive protein, Alb: Albumin, FBS: Fasting blood glucose, eGFR: estimated glomerular filtration rate, Hct: Hematocrit.
