# Supplementary material for: Serum Uric Acid and Chronic Kidney Disease: The Role of Hypertension
Source: PLoS One. 2013 Nov 12;8(11):e76827. doi: 10.1371/journal.pone.0076827 (PMC3827035; doi:10.1371/journal.pone.0076827)
Supplement: Table S2 — Comparing baseline characteristic of population for analysis with missing population (died or loss to follow up). (DOCX) [file pone.0076827.s003.docx]

**Table S2:** Comparing baseline characteristic of population for analysis with missing population (died or loss to follow up)

|  | **Population for analysis**  **(n=2601)** | **Missing population**  **(n=2374)** | **P-value** |
| --- | --- | --- | --- |
| **Age, mean (SD*), y** | 66.4 (6.9) | 74.5 (9.4) | <0.001 |
| **Men, n (%)** | 1009 (38.8) | 864 (36.4) | 0.084 |
| **Daily Alcohol Intake in drinkers, median (Interquartile range), g/d** | 3.8 (0.3-14.9) | 2.1 (0.0-13.5) | <0.001 |
| **Body mass index, mean (SD), kg/m^2^** | 26.3(3.5) | 26.1 (3.9) | <0.001 |
| **Total cholesterol, mean (SD), mmol/L** | 6.7 (1.2) | 6.5 (1.2) | <0.001 |
| **HDL cholesterol, mean (SD), mmol/L** | 1.3 (0.3) | 1.3 (0.3) | 0.988 |
| **C-reactive protein, median**  **(Interquartile range), mg/L** | 1.5 (0.8-3.0) | 2.3 (1.1-4.4) | <0.001 |
| **Glomerular filtration rate, mean (SD),**  **ml/min per 1.73 m2** | 80.1 (15.4) | 73.9 (18.5) | <0.001 |
| **Systolic blood pressure, mean (SD), mm Hg** | 135.3(20.0) | 143.1 (23.5) | <0.001 |
| **Diastolic blood pressure, mean (SD), mm Hg** | 73.3 (10.8) | 73.1 (12.5) | <0.001 |
| **Chronic kidney disease, n (%)** | 196 (7.5) | 489 (20.6) | <0.001 |
| **Diabetes Mellitus, n (%)** | 192 (7.4) | 356 (15.2) | <0.001 |
| **History of coronary heart disease, n (%)** | 279 (10.8) | 387 (16.7) | <0.001 |
| **Diuretics, n (%)** | 285 (11.0) | 543 (22.9) | <0.001 |
| **Calcium channel blockers, n (%)** | 123 (4.7) | 181 (7.6) | <0.001 |
| **Beta-blockers, n (%)** | 379 (14.6) | 343 (14.4) | 0.904 |
| **ACE inhibitors, n (%)** | 98 (3.8) | 163 (6.9) | <0.001 |
| **Serum uric acid, mean (SD), mg/dL**** | 5.2 (1.2) | 5.5 (1.4) | <0.001 |

*****SD: Standard deviation

**To convert to SI unit multiply by 59.48
